# The effect of immunization schedule with the malaria vaccine candidate RTS,S/AS01_E_ on protective efficacy and anti-circumsporozoite protein antibody avidity in African infants

**DOI:** 10.1186/s12936-015-0605-7

**Published:** 2015-02-13

**Authors:** Anthony Ajua, Bertrand Lell, Selidji Todagbe Agnandji, Kwaku Poku Asante, Seth Owusu-Agyei, Grace Mwangoka, Maxmilliam Mpina, Nahya Salim, Marcel Tanner, Salim Abdulla, Johan Vekemans, Erik Jongert, Marc Lievens, Pierre Cambron, Chris F Ockenhouse, Peter G Kremsner, Benjamin Mordmüller

**Affiliations:** Eberhard Karls Universität Tübingen, Institut für Tropenmedizin, Wilhelmstraße 27, 72074 Tübingen, Germany; Centre de Recherches Médicales de Lambaréné (CERMEL), BP118 Lambaréné, Gabon; Kintampo Health Research Centre, PO Box 200, Kintampo, Ghana; Faculty of Infectious and Tropical Diseases, London School of Hygiene and Tropical Medicine, Keppel Street, London, WC1E 7HT UK; Bagamoyo Research and Training Centre of Ifakara Health Institute, Bagamoyo, 360 Kiko Avenue, Mikocheni, PO Box 78373, Dar es Salaam, Tanzania; Swiss Tropical and Public Health Institute, Basel, Switzerland; GlaxoSmithKline Biologicals, Rixensart, Belgium; PATH Malaria Vaccine Initiative, 455 Massachusetts Avenue NW, Suite 1000, Washington, DC 20001 USA

**Keywords:** Malaria, RTS,S, Vaccine, *Plasmodium falciparum*, Antibody, Avidity, Correlate of protection

## Abstract

**Background:**

The malaria vaccine RTS,S induces antibodies against the *Plasmodium falciparum* circumsporozoite protein (CSP) and the concentration of Immunoglobulin G (IgG) against the repeat region of CSP following vaccination is associated with protection from *P. falciparum* malaria. So far, only the quantity of anti-CSP IgG has been measured and used to predict vaccination success, although quality (measured as avidity) of the antigen-antibody interaction shall be important since only a few sporozoites circulate for a short time after an infectious mosquito bite, likely requiring fast and strong binding.

**Methods:**

Quantity and avidity of anti-CSP IgG in African infants who received RTS,S/AS01_E_ in a 0-1-2-month or a 0-1-7-month schedule in a phase 2 clinical trial were measured by enzyme-linked immunosorbent assay. Antibody avidity was defined as the proportion of IgG able to bind in the presence of a chaotropic agent (avidity index). The effect of CSP-specific IgG concentration and avidity on protective efficacy was modelled using Cox proportional hazards.

**Results:**

After the third dose, quantity and avidity were similar between the two vaccination schedules. IgG avidity after the last vaccine injection was not associated with protection, whereas the change in avidity following second and third RTS,S/AS01_E_ injection was associated with a 54% risk reduction of getting malaria (hazard ratio: 0.46; 95% confidence interval (CI): 0.22-0.99) in those participants with a change in avidity above the median. The change in anti-CSP IgG concentration following second and third injection was associated with a 77% risk reduction of getting malaria (hazard ratio: 0.23, 95% CI: 0.11-0.51).

**Conclusions:**

Change in IgG response between vaccine doses merits further evaluation as a surrogate marker for RTS,S efficacy.

**Trial registration:**

ClinicalTrials.gov Identifier NCT00436007.

## Background

Malaria has an enormous public health impact and new preventive interventions are urgently needed. After more than 100 years of research on malaria vaccines, RTS,S was the first pre-erythrocytic vaccine candidate that entered phase III clinical development [1-3]. RTS,S contains hepatitis B surface antigen (HBsAg) together with a fusion protein of HBsAg and a carboxy-terminal fragment of *Plasmodium falciparum* circumsporozoite protein (CSP), co-expressed in yeast and formulated with a proprietary adjuvant (AS01). The exact mechanism of RTS,S-mediated protection is not known, although Immunoglobulin G antibodies (IgG) against the CSP repeat region are likely to play an important role since the concentration of anti-CSP IgG partly explains protection in most studies that assessed efficacy of RTS,S in African children [4-6]. In addition, passive transfer of anti-CSP IgG can protect animals from subsequent challenge [7,8]. Besides concentration, many other properties determine antibody function. Among them are availability of effector molecules, post-translational modification, isotype, subclass, affinity and avidity of antibodies. It is difficult to measure all these characteristics in one sample, particularly in the small sample volumes obtained during clinical trials in infants. Affinity, defined as the strength of interaction between an epitope and an antibody binding site, would be a particularly interesting variable to measure in the context of anti-CSP IgG-mediated immunity, since the time of interaction with the parasite is short (less than 30 minutes [9]), sporozoites are strongly diluted and few. In fact, only one successful hepatocyte infection is sufficient to initiate and maintain blood stage infection. Studies in mice have shown that high antibody affinity against a synthetic CSP immunogen is positively associated with protection [8,10] and most studies in humans indicate that anti-CSP IgG concentration explains only parts of the vaccine-mediated protection. Increase in antibody affinity after repeated antigen exposure is the result of affinity maturation due to somatic hypermutation. The rate and extent of maturation may be influenced by several factors, including nature, route and dose of the antigen, adjuvants and carriers as well as the immunization schedule. In the present study antibody avidity was measured. It is a representation of the strength of interaction between antibodies and antigens in a complex and besides antibody affinity, valences of antibodies and antigens as well as structural features of the complex are important determinants of avidity. For CSP, it has been shown that the use of some adjuvants can increase the avidity of anti-CSP IgG after vaccination of human volunteers [11]. In this study IgG avidity against the repeat region of CSP was measured after the second and third injection of RTS,S/AS01_E_ in infants that received the vaccine as part of a phase IIb clinical trial to assess safety and efficacy of RTS,S/AS01_E_ in the age-group targeted by the expanded programme on immunization (EPI) [5,12].

## Methods

### Clinical trial

The objective of the study was to explore the effect of anti-CSP IgG avidity on RTS,S vaccine efficacy in naturally exposed infants. Details of the clinical trial have been published previously [5,12]. Briefly, safety and efficacy of RTS,S/AS01_E_ when given through the EPI was assessed in 511 children from Gabon, Ghana and Tanzania. Participants were randomly assigned to one of three intervention arms: 1) RTS,S/AS01_E_ as three injections, one month apart (0, 1, 2 months schedule [012]; n = 170), 2) RTS,S/AS01_E_ extended schedule (0, 1, 7 months schedule [017]; n = 170) or 3) control (EPI vaccines alone; n = 171). Malaria was defined as parasitaemia >500 parasites per μl and an axillary temperature >37°C. The efficacy of RTS,S against first malaria episodes, detected by passive case detection, was equivalent in the two schedules one year after the third injection. The study followed Good Clinical Practice guidelines, the Declaration of Helsinki (4^th^ revision) and received approval from the appropriate local and national ethics committees of each site. In addition, ethical review by the ethics committees of the London School of Hygiene and Tropical Medicine Ethic Committee, the Swiss Tropical Institute Committee and the Western Institutional Review Board was sought. The trial is registered with ClinicalTrials.gov (NCT00436007).

### Antibody measurements

Antibodies against CSP were measured by evaluating IgG responses against the CSP-repeat region, using a validated enzyme-linked immunosorbent assay (ELISA) with R32LR as the coating antigen [13]. An anti-CSP IgG titre of 0.5 ELISA units per millilitre (EU/mL) or greater was considered to be positive. For measurements of avidity of IgG against the repeat region of CSP, samples were evaluated as described [13], but in two different plates; one treated with a chaotropic agent and one untreated plate. As chaotropic agent a 1 M solution of ammonium thiocyanate (NH_4_SCN) was added in the treatment plate while 0.05% Tween-20 in PBS was added in the untreated plate and both CSP ELISA plates were further washed and developed as described [13]. The avidity index (AI) was calculated as the ratio of the concentration of anti-CSP IgG (EU/ml) that remained bound to the coated antigen after treatment with NH_4_SCN, divided by the concentration of IgG (EU/ml) that remained bound to the coated antigen in the untreated plate. Anti-CSP IgG quantification and avidity were measured at the Center for Vaccinology, Ghent University Hospital, Belgium.

For statistical modelling the logarithm of anti-CSP IgG concentration was used since previous data showed that log-transformation results in a better fit to the normal distribution. AI was analysed in the two RTS,S-vaccinated arms and after the second and third vaccination. Since the majority of infants before vaccination and those receiving control vaccine do not have measurable anti-CSP IgG, AI cannot be calculated. Delta AI (dAI) was defined as the difference in AI between the second and third vaccination. Similarly, delta CSP (dCSP) was defined as the difference in anti-CSP IgG concentration between the second and third vaccination.

### Statistics

Analysis of the effect of IgG avidity on protective efficacy was exploratory and not detailed in the statistical analysis plan of the original study. IgG responses between the groups were analysed by descriptive statistics and represented as boxplots together with the individual measurements. The effect of anti-CSP IgG concentration and AI on risk of malaria was calculated using the according-to-protocol (ATP) dataset with a Cox proportional hazards model in R v2.15.2. For statistical modelling antibody concentrations were log-transformed. To calculate the effect of dAI and dCSP on the occurrence of malaria episodes with a Cox proportional hazards model, values were dichotomized on the median dAI or dCSP and labelled as ‘high’ and ‘low’, respectively. All models included the covariates schedule and site. If appropriate, other covariates were added as reported in the results section. A p-value below 0.05 was considered significant and 95% confidence intervals (95% CI) are given where appropriate.

## Results

**Figure 1:**
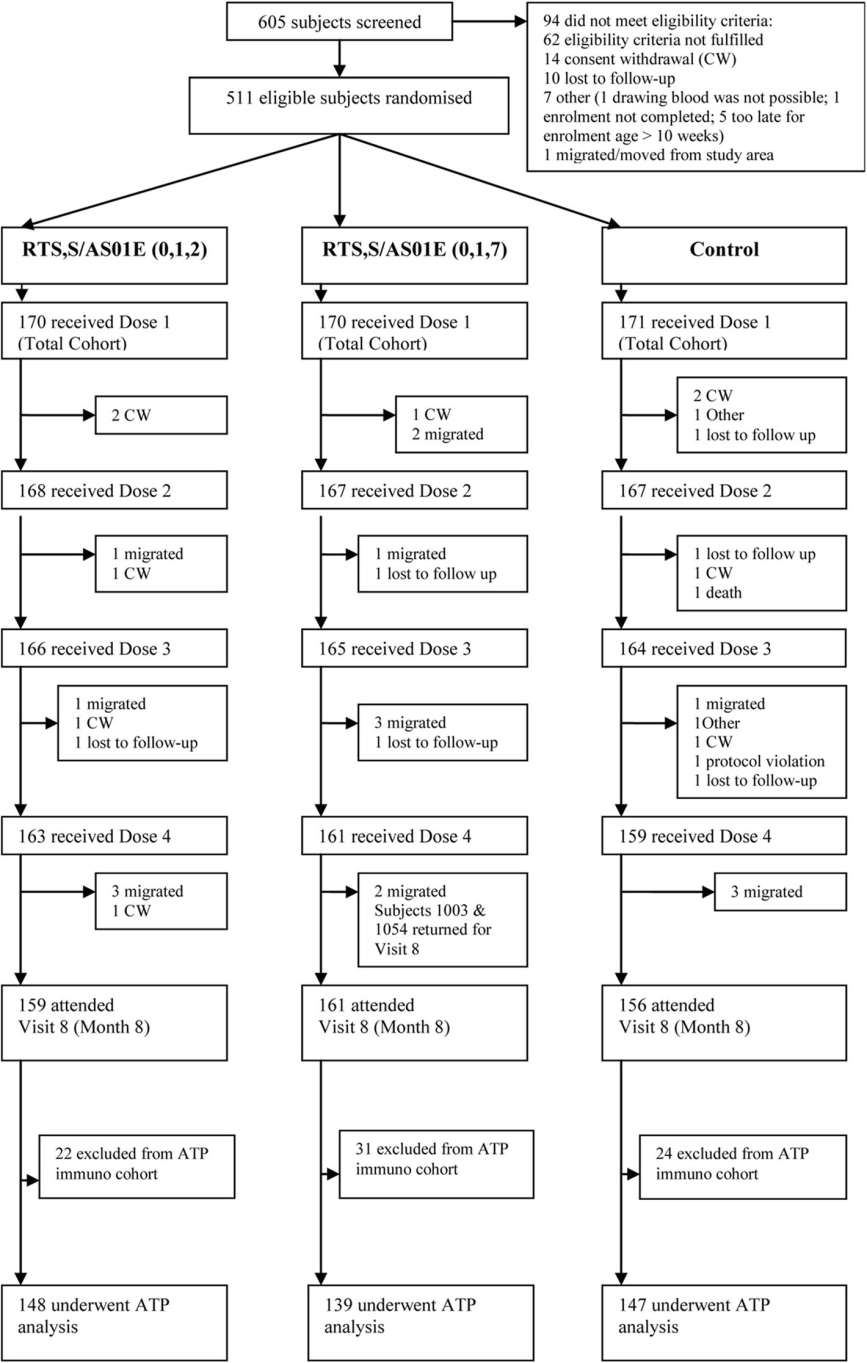
**CONSORT study flow chart.**

After screening 605 participants, 170 received RTS,S in the standard (012) and 170 in the extended (017) schedule, as depicted on the CONSORT flowchart of the primary study (Figure 1). Samples from 315 (300 ATP) participants were available for immunological analysis (012: n = 154 [148]; 017: n = 161 [152]). Paired immunological samples to calculate dAI were available from 187 (179 ATP) participants (012: n = 103 [100]; 017: n = 84 [79]).

As reported earlier [5], high anti-CSP IgG titres after three vaccine injections were associated with a reduction in subsequent incidence of clinical malaria: the hazard ratio of a ten-fold increase in anti-CSP IgG was 0.52 (95% CI: 0.34-0.81), which corresponds to a 48% risk reduction.

**Figure 2:**
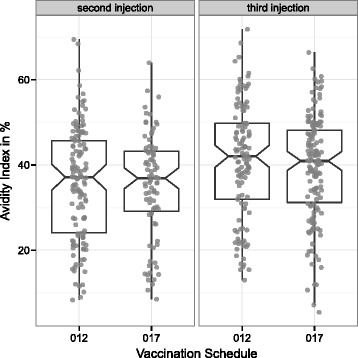
**Box-plot and single measurements of absolute AI at second and third vaccination using two vaccination schedules (012 or 017).**

**Figure 3:**
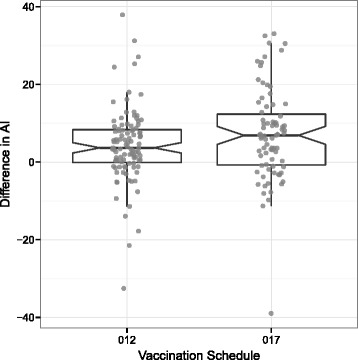
**Box-plot and single measurements of difference in AI (dAI) between second and third vaccination using two vaccination schedules (012 or 017).** Note that for the analysis of dAI only paired samples were used (n = 179).

Absolute AI after two (012: 35.9, 017: 34.9; t-test p = 0.57) and three (012: 41.2, 017: 39.3; t-test p = 0.22) RTS,S injections were similar between the two vaccination schedules (Figure 2). As expected, an increase in AI between the second and third vaccination was present (Figure 3). Increase in delta AI (dAI) was slightly, albeit not statistically significant, higher in the 017 (7.1) group compared to the 012 (4.2) group (delta: 3.0; 95% CI: −0.3-6.1; t-test p = 0.08).

**Figure 4:**
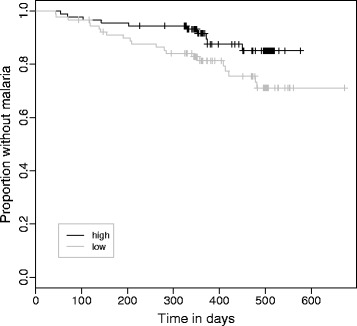
**Kaplan Meier plot of malaria episodes over time in participants classified as having high (black) or low (grey) dAI.**

To explore the effect of AI, dAI and dCSP on malaria risk, three Cox proportional hazard models were defined and tested. AI after the third injection, corrected for site, schedule and anti-CSP IgG concentration, did not explain a significant reduction in risk of clinical malaria (Model 1; hazard ratio: 0.99, 95% CI: 0.97-1.02). Participants were then divided on the median in dCSP and dAI ‘high’ and ‘low’ responders and included as categorical variable in the model. Classification as ‘high-dCSP’ was associated with a significant risk reduction (77%) compared to the ‘low dCSP’ group in a model corrected for site and schedule (Model 2; hazard ratio: 0.23, 95% CI: 0.11-0.51). When dAI, corrected for site, schedule and dCSP was analysed, the hazard ratio between high and low responders separated by the median, was 0.46 (Model 3; 95% CI: 0.22-0.99; Wald test p = 0.049), hence classification as ‘high dAI’ group member is associated with a 54% risk reduction (Figure 4).

## Discussion

The complex interplay of vaccine-primed immune mediators that define a successful response upon pathogen encounter is not well understood. Cellular and humoral components have important roles, although in various compositions, depending on the pathogen and the host. Antibodies are the prototypic vaccine-induced immune mediators and play an important role in anti-malarial immunity during the pre-erythrocytic [8,10] as well as the erythrocytic stage [14] of the disease, as shown by passive transfer experiments in mice and man. The sheer concentration of antigen-specific antibodies is normally used to measure immunization success and serves as a surrogate to estimate protective efficacy. The clinical development of RTS,S is a unique opportunity to investigate the effect of further variables such as antibody avidity, isotype or subclass on vaccine efficacy, since clinical (true) efficacy is known [5], being 57% (95% CI: 33–73) with the 012 schedule and 32% (95% CI: 16–45) following the 017 schedule.

Here, anti-CSP IgG avidity was measured to assess if it predicts vaccine efficacy in a phase II clinical trial of RTS,S independent of anti-CSP IgG concentration [5,12]. Regardless of the vaccination scheme and site, avidity did not improve prediction over anti-CSP IgG concentration alone. This may mean that: i) the assay is not sensitive enough to reflect avidity; ii) collinearity between antibody concentration and avidity blurs the effect of avidity; or, iii) that avidity is not an important determinant of vaccine efficacy. In this study IgG concentration and avidity was measured after the second and third vaccine injection. This approach is valid to assess if the immune system reacted to vaccination successfully. Since kinetics of IgG vary over time and the study was performed under natural exposure to malaria parasites, the time of encounter with the parasite becomes an important variable. This is in contrast to controlled human malaria infection (CHMI) studies, where the time of infection is defined. Hypothetically, the difference in IgG concentration (and avidity) between second and third vaccination could be a better predictor of effective antibody-mediated protection than concentration after the third vaccine injection, because it better reflects the further evolution of antibody responses until next parasite encounter. The present data argue for the use of this approach since it was shown that a high dCSP predicts protective efficacy and dAI explains part of the protection in the RTS,S vaccinated children (Model 3). How AI evolves over time and if it is a useful predictor of vaccine efficacy remains to be validated with further, independent and confirmatory studies.

Nevertheless, this observation adds a new component to the search of correlates of protection and the understanding of the immune responses elicited by pre-erythrocytic malaria vaccine candidates such as RTS,S. Since adjuvants also have a profound effect on the speed of avidity maturation [11], the effect of avidity on vaccine efficacy could even be analysed with interventional studies that assess the effect of timing between immunizations (as in this study) and different adjuvants on protective efficacy while direct measures of maturation of the immune system such as single-cell based sequencing of IgG genes of anti-CSP memory B-cells [15,16] are performed. This may be particularly interesting for antigens such as CSP that are not highly immunogenic *per se*, because highly immunogenic antigens often induce antibodies with strong avidity over a short period of time and a threshold antibody concentration is appropriate to predict their efficacy [17]. Other studies in the development of RTS,S (e.g., challenge experiments [18] and the recently completed phase III trial [1-3]) will certainly provide additional information and may establish the measurement of avidity as one biomarker for vaccine efficacy. Additionally, such knowledge may guide the design of next generation vaccines and administration schemes.

## Conclusions

So far, the most robust correlate of protection for the malaria vaccine candidate RTS,S is anti-circumsporozoite (CSP) IgG concentration following immunization. Pre-clinical data and theoretical considerations suggest that avidity may have an additional impact on protective efficacy. It is shown that an increase in anti-CSP IgG concentration and avidity between second and third vaccine injection is associated with a strong risk-reduction for malaria after immunization. This finding shall influence the way of analysis of immunological correlates of protection since using change in antibody concentration and avidity rather than single measurements enables improved modelling of immune-effector function at the time of pathogen encounter and hence more powerful prediction of vaccine efficacy.

### Consent

Written informed consent was obtained from each child’s parent(s). Illiterate parents were informed about the study in the presence of an impartial and literate witness and informed consent was documented by thumbprint of the parent and signature of the witness.

## Abbreviations

AI: Avidity index
dAI: delta AI
CSP: *Plasmodium falciparum* circumsporozoite protein
dCSP: Delta CSP
ATP: according to protocol
IgG: Immunoglobulin G
